# Habitat Use and Body Mass Regulation among Warblers in the Sahel Region during the Non-Breeding Season

**DOI:** 10.1371/journal.pone.0113665

**Published:** 2014-11-26

**Authors:** James O. Vafidis, Ian P. Vaughan, T. Hefin Jones, Richard J. Facey, Rob Parry, Robert J. Thomas

**Affiliations:** 1 Cardiff School of Biosciences, Cardiff University, Wales, CF10 3AX, United Kingdom; 2 Wildlife Trust of South and West Wales, Parc Slip, Tondu, Bridgend, CF32 0EW, United Kingdom; Liverpool John Moores University, United Kingdom

## Abstract

Migratory birds face significant challenges across their annual cycle, including occupying an appropriate non-breeding home range with sufficient foraging resources. This can affect demographic processes such as over-winter survival, migration mortality and subsequent breeding success. In the Sahel region of Africa, where millions of migratory songbirds attempt to survive the winter, some species of insectivorous warblers occupy both wetland and dry-scrubland habitats, whereas other species are wetland or dry-scrubland specialists. In this study we examine evidence for strategic regulation of body reserves and competition-driven habitat selection, by comparing invertebrate prey activity-density, warbler body size and extent of fat and pectoral muscle deposits, in each habitat type during the non-breeding season. Invertebrate activity-density was substantially higher in wetland habitats than in dry-scrubland. Eurasian reed warblers *Acrocephalus scirpaceus* occupying wetland habitats maintained lower body reserves than conspecifics occupying dry-scrub habitats, consistent with buffering of reserves against starvation in food-poor habitat. A similar, but smaller, difference in body reserves between wet and dry habitat was found among subalpine warblers *Sylvia cantillans* but not in chiffchaffs *Phylloscopus collybita* inhabiting dry-scrub and scrub fringing wetlands. Body reserves were relatively low among habitat specialist species; resident African reed warbler *A. baeticatus* and migratory sedge warbler *A. schoenobaenus* exclusively occupying wetland habitats, and Western olivaceous warblers *Iduna opaca* exclusively occupying dry habitats. These results suggest that specialists in preferred habitats and generalists occupying prey-rich habitats can reduce body reserves, whereas generalists occupying prey-poor habitats carry an increased level of body reserves as a strategic buffer against starvation.

## Introduction

Migratory birds face the challenge of finding sufficient food resources on their wintering grounds to avoid starvation during the non-breeding season and to prepare themselves for the return migration to their breeding grounds. To do this, they must select suitable wintering habitat, in competition with other migrant and resident species [1]–[3]. The causes and consequences of habitat choices made by migratory birds outside the breeding season are widely studied and much debated [4]–[7]. Although it is well established that competition affects the distribution of individuals on their temperate breeding grounds [8]–[9], it has been suggested that competition may be less important on the wintering grounds where migrant birds may have lower energetic requirements [10]–[12]. Migratory birds arriving *en masse* in the wintering areas must, however, select between habitats that may differ in foraging quality, the density of resident and migrant competitors, and other factors such as predation pressure [13], [14]. The strength of these factors is likely to be affected by variations in climate. Indeed, overwinter survival of some long-distance migrant birds is closely linked to climate-driven measures of broad-scale environmental conditions on their wintering grounds, such as El Niño Southern Oscillation, Sahel Precipitation Index and Normalised Difference Vegetation Index [15]–[20]. Such correlations imply that survival of some, or many, migrant species may be limited by overwinter foraging conditions.

In a competition-driven system, residents and dominant or early-arriving migrants may be able to secure territories in primary (higher quality) habitat while subordinate or late-arriving individuals may be competitively excluded into secondary habitats: the competitive exclusion hypothesis [18], [21], [22]. Compelling evidence for the role of inter-specific competitive exclusion in the shaping of migrant and resident communities in the wintering grounds is lacking [2], [10], [12]. However, evidence for such competitive exclusion in an intra-specific context was provided by Perez-Tris and Telleria (2002) [23], who showed that resident blackcaps *Sylvia atricapilla* breeding in southern Spain maintained their breeding territories in primary forest habitats into the non-breeding season, while migrant blackcaps arriving at the start of the wintering season ‘leap-frogged’ into the secondary (lower quality) scrub habitats not occupied by residents.

An alternative to the competitive exclusion hypothesis (though not necessarily mutually exclusive with it) is that migrants are “eurytopic” (i.e. adaptable) and have less restrictive habitat requirements during the non-breeding season, allowing them to use a wider range of winter habitat types than ecologically similar but more specialised resident species [2], [21], [24]. Such adaptability and breadth of foraging strategies has been shown in wintering golden orioles *Oriolus oriolus*, which were, by virtue of their wider range of foraging tactics, able to survive in lower quality microhabitats than were preferred by resident species [25]. Migrants may therefore be able to exploit lower quality habitats with lower densities of resident competitors [26]–[29], even though they may be markedly different from the habitat types used on their temperate breeding grounds [12], [24], [30], [31]. Furthermore, migrants may be able to occupy habitats which residents may find unsuitable, either due to the vegetation structure or insufficient food availability to sustain future breeding attempts (i.e. habitats of ‘low breeding currency’; [32], [33]).

It is well-established by both theoretical and empirical studies that the level of body reserves carried by small birds reflects a trade-off between starvation and mass dependent costs of body reserves [34], [35]. These mass dependent costs include increased predation risk, flight costs and increased foraging requirements [36]–[39]. Balanced against these mass-dependent costs are the benefits of the stored body reserves acting as a strategic buffer against starvation in situations where food is scarce or unpredictable. This hypothesis of ‘adaptive mass-regulation’ or ‘strategic buffering against starvation’ is supported by experimental studies on north-temperate wintering birds such as greenfinches *Carduelis chloris* [40], European starlings *Sturnus vulgaris* [41], [42] and great tits *Parus major* [43]. These species responded to food limitation by accumulating mass (in comparison to controls) and responded to food supplementation by decreasing mass. Strategic buffering has been implicated in the observed fattening of wintering brambling *Fringilla montifringilla* [44], great tits [45] and Siberian Jays *Perisoreus infaustus* [46] in response to declining food availability or the risk of prolonged periods of food limitation. While the influence of predation pressure and competition are important determinants of energy reserves carried by birds [39], [47], it is clear that the overall availability of food in a habitat has a major influence on bird foraging and mass regulation strategies. Despite these examples, however, little is known about the mass regulation strategies used by European migrants wintering in Africa.

Wetlands in the western Sahel, mostly within the Inner Niger Delta and Senegal Delta, represent the main overwintering area for many Western Palearctic-African migrant bird species [48]. The area and quality of these wetlands is dependent on the extent of summer rainfall across West Africa. These gradually declined through much of the 20^th^ Century [5], reducing their carrying capacity for wintering songbirds [16], [17]. In addition to climate impacts, habitat loss and degradation from agriculture and irrigation have led to further reduction of available wetland habitat in the Sahel [5]. Many of the areas surrounding the wetlands comprise dry scrub habitats, which are also occupied by migrants.

The invertebrate-rich wetlands support a mixed community of insectivorous ‘Old World’ warblers (superfamily *Sylvoidea*), including the resident species African reed warbler *Acrocephalus baeticatus* and greater swamp warbler *A. rufescens*, as well as migrant species including sedge warbler *A. schoenobaenus* and Eurasian reed warbler *A. scirpaceus* [49]–[53]. The dry scrub supports dry-habitat breeding migrant warblers, such as subalpine warbler *Sylvia cantillans*, western olivaceous warbler *Iduna opaca* and common chiffchaff *Phylloscopus collybita* (henceforth “chiffchaff” [54]). Most of these species are restricted to either wetland or scrub habitats, but three are relative habitat generalists: Eurasian reed warbler regularly occupies dry scrub, while subalpine warbler and chiffchaff often forage in the ecotone between scrub and wetland habitats. Such use of multiple winter habitat types by warbler species is well known [2], [55], [56]. Furthermore, there appears to be significant intra-specific variation in the body reserves (in the form of fat and pectoral muscle) of birds wintering in West Africa [57], [58], although the reasons for such variation are unknown. However, while several studies have shown intra-specific responses of wintering migrant birds to habitat quality gradients in Neotropical ecosystems [59], [60], [20], no study has attempted to investigate mass regulation strategies among warbler species occupying the two major types of wintering habitats in the Sahel; wetland and dry-scrub.

In the present study, we investigated the distribution, structural size and extent of body reserves (in the form of fat and pectoral muscle) of insectivorous songbirds in a wintering area in Senegal, West Africa, during three successive non-breeding seasons. The aims of the study were to: 1) assess the relative abundance of invertebrate prey resources in wetland and dry scrub habitats, providing a measure of habitat quality; 2) identify habitat preferences of five migratory warbler species and one resident, comprising two wetland specialists, one dry scrub specialist and three habitat generalists; and 3) compare the body mass, fat, pectoral muscle and size of individuals in wet and dry habitats, to test the hypothesis that habitat usage and body condition are driven by strategic responses to the availability of food in the different habitats. We tested predictions about the level of body reserves (in the form of fat and pectoral muscle) expected under conditions of direct food limitation compared to strategic regulation of body reserves, in both wetland and dry-scrub habitats. Specifically, if warblers are strategically regulating their body reserves as a buffer against starvation, we predict that individuals inhabiting dry scrub habitat where food availability is lower (and thus starvation risk is greater), carry higher levels of energy reserves than in habitats where food is more abundant. If, however, warbler body reserves are directly limited by food availability, we predict body reserves to be lower in the habitat with lowest food availability. By including the ‘habitat specialist’ species African reed warbler, sedge warbler and western olivaceous warbler in this study, we test whether this pattern extends across species among habitat specialist and habitat generalist species differing in their migratory tendency and timing of arrival on the wintering grounds.

## Methods

### Ethics Statement

The guidelines promoted by the Association for the Study of Animal Behaviour for the ethical use of animals in research were followed. All species caught as part of this study are common and not registered as an endangered or protected species in any country. All fieldwork was conducted after ethical approval from Parc National des Oiseaux du Djoudj (permit number 021 403) and the British Trust for Ornithology ringing unit (Vafidis license 5475; Facey license 5411).

### Study Area

The ‘Parc National des Oiseaux du Djoudj’ (16° 21′ 59″ N, 16° 16′ 26″ W) is located in the semi-arid zone in Senegal, West Africa and covers an area of 16,000 ha of seasonally flooded waterways. Its landscape is *Acacia-Commiphora* grassland and *Tamarix senegalensis* scrub savannah interspersed with a range of *Phragmites* and *Typha*-dominated wetland habitats. The study area supports wintering populations of a range of warbler species including Eurasian reed warbler, sedge warbler, chiffchaff, subalpine warbler, western olivaceous warbler and African reed warbler (Table 1). Fieldwork was conducted near the village of ‘Diadiem 3’ (16° 21′ 7″ N, 16° 16′ 34″ W), in the south west of the national park in wetland and dry scrub habitats near Marigot du Khar. Permission to undertake fieldwork was obtained from the national park authority.

**Table 1:** Study species status and winter habitat preferences.

Study Species	Status	Winter Habitat
*Eurasian reed warbler*	migrant	generalist
*common chiffchaff*	migrant	generalist
*subalpine warbler*	migrant	generalist
*sedge warbler*	migrant	wetland
*African reed warbler*	resident	wetland
*western olivaceous warbler*	migrant	dry scrub

We used mist nets to sample the community of warblers occupying four locations within an approximately 40 hectare section of wetland (centred at 16° 22′ 25″ N, 16° 16′ 12″ W) over three winter seasons (January 2012, 2013 and 2014) and six locations within an approximate 400 hectare section of dry scrub habitat (centred at 16° 21′ 44″ N, 16° 15′ 49″ W) in the surrounding area in two winter seasons (January 2013 and 2014). Wetland study sites were located 260–1550 m apart. Dry scrub study sites were located 100–2000 m apart. The minimum distance between wetland and dry study sites was 775 m. Although individual birds were occasionally caught in sites adjacent to those in which they were originally ringed, no individuals ringed in wetland sites were recorded in dry scrub sites, or vice-versa. Bird biometric data were collected during 24 morning or evening ringing sessions across the three years, during a period when birds were not migrating (between 17 and 29 January in each year). Each habitat type was subject to the same netting effort per ringing session, over the course of the study (66 m per session, approximately 36 hr/m). Nets were checked at least every 20 minutes.

### Bird biometric data

Captured birds were taken in cotton bags to a nearby processing station where species and age (where possible) were recorded following Svensson (1992)[61]. The following biometric measurements taken: wing length (maximum wing chord to 1 mm), tarsus length (tarsal joint to top of flattened foot to 0.1 mm), total head length (back of skull to distal tip of bill, to 0.1 mm) and mass using an electronic balance (to 0.1 g) with time of weighing recorded. The measurement techniques followed the methods described by Svensson [61]. The size of the pectoral muscle was scored following Kaiser (1993)[62] (0 =  emaciated to 3 =  large muscle mass) and subcutaneous fat deposits were estimated following Bairlein (1995 [63]: fat score; 0 =  no visible subcutaneous fat, 8 =  whole belly covered in fat). To eliminate among-observer variability, all measurements were either made or checked by one person (JV). The recording period occurred after the typical winter moulting period for Eurasian reed and sedge warblers, so age was generally not determinable. Each bird was fitted with a British Trust for Ornithology (BTO) issued metal ring.

### Invertebrate Monitoring

A measure of invertebrate prey availability was determined using sticky traps (yellow, double sided, effective area 100 cm^2^, Oecos, Hertfordshire, UK). These traps are highly effective for sampling the activity-density of Diptera [64]–[66], the primary prey taxon of Eurasian reed warbler in Europe (R.A. King, personal communication). To assess stability of wetland invertebrate prey resources over the dry season we monitored the invertebrates on a weekly basis for a period of three months between 16 January and 20 March 2012. Seven traps were set in each of the four sites used for bird monitoring, attached to a mixture of scrub and Phragmites vegetation at heights of between 0.5–1.2 m and at least 10 m away from mist net positions and any regular pathways used by mammals (including humans). These wetland sites and four dry scrub habitats were also monitored using seven traps in each site over the course of one week in 2013 and 2014 (with traps set in the same positions in wetlands as in 2012) and in dry scrub habitats. Invertebrates were not monitored in dry scrub habitats in 2012. Traps were attached around the edges of habitat fragments set at heights between 0.5–1.2 m.

Total captures of Diptera, Arachnidae, Hymenoptera and Hemiptera, as well as other less-frequently encountered taxa (<1%) such as Coleoptera and Lepidoptera recorded in each habitat, providing a cumulative measure of activity-density, were compared between wetland and dry scrub sites. To assess general differences in invertebrate size between habitats, all sampled invertebrates were measured for body length (excluding legs, wings and antennae) and categorised as either small (≤5 mm) or large (>5 mm). This threshold size value is used in other similar studies testing for prey differences for wintering small billed migrant birds for which diet is primarily comprised of small invertebrate prey [33], [67]. The difference in distribution of invertebrate size (small and large) was compared between wetland and dry scrub sites. Mean daily temperatures were recorded in each habitat type using temperature loggers (LASCAR EL USB-1).

### Data analysis

All analysis was undertaken using R version 3.0.3. [68].

### Assessment of Invertebrate Resources

1. 2012 Wetland habitat analysis

The invertebrate activity-density across the 2012 study period were analysed with generalized linear mixed-effects models (GLMM; R package “lme4” [69]) using date as a fixed effect and trap location as a random intercept.

1. 2013 and 2014 between-habitat analysis

Differences in activity-density and the distribution of invertebrate size using the January 2013 and 2014 invertebrate data were analysed using a generalized linear model (GLM) using habitat type (“wetland” or “dry scrub”) and year as factors. Differences in the variances of invertebrate samples between habitat types were tested using an *F*-test.

### Assessment of habitat preferences

We assessed habitat preference among warbler species by modelling probability of occurrence using a binomial GLM with ‘habitat’ as the binary dependant variable (i.e. “dry” or “wet”), and species as an independent variable. Habitat preferences were compared among species using the ‘contrast’ package [70], with positive and negative parameter values representing closer association with wetlands and dry scrub habitats, respectively.

### Body mass, muscle, fat and body size comparisons

Intra-specific comparisons of body mass and structural body size (wing length) were made for birds occupying both habitats. Body mass and structural body size was not comparable between species because of differences in overall size and shape of species. Comparisons of fat and muscle reserves of birds using both habitat types were made between species as well as within species.

Body mass was compared between habitats for each generalist species (Eurasian reed warbler, subalpine warbler and chiffchaff) using a generalised additive model (GAM) implemented using the “mgcv” package [70]. Habitat type was modelled as a factor, flattened wing chord, total head length and tarsus length were modelled as linear relationships to control for body size, and time of day was modelled using a cyclic cubic regression spline to allow for the diurnal variation in body mass [71]. We also tested for differences in mass between years, using the same model structure, but with year added as a fixed factor. For birds captured on more than one occasion during the same winter, only the first capture event was included in the analysis, to avoid pseudo-replication. Only six birds were captured in more than one winter, and their recaptures in subsequent years were excluded from the analysis. Residual diagnostic plots from the models were used to verify the assumptions of normality and homogeneity of model residuals, and to test for unduly influential observations [72].

Intra-specific differences in the structural body size of individuals between habitat types were compared using a general linear model (GLM), using flattened wing chord as a dependent variable, and habitat type and year as independent effects. An alternative model using the first principal component (PC1) of a principal components analysis (PCA) of the three measures of bird body size (flattened wing chord, tarsus and total head length) as a dependant variable did not improve the fit of the model.

Inter- and intra-specific comparisons of body condition were conducted using muscle and fat scores, since these scores represent an index of energy reserves and allows for direct comparisons of condition between species of different size and morphology (such comparisons using body mass are problematic because of the difficulty in defining lean body mass in live birds). Comparisons between species of fat and muscle reserves are valid because these are size-independent variables [73]–[76]. Since the muscle and fat score methods applied in this study used ordered discrete values (0–3) and (0–8) respectively, a proportional odds regression (a special case of ordinal logistic regression) was used to compare muscle and fat scores between habitats, while controlling statistically for time of day [77]. Proportional odds regression models were fitted as ordinal regression models, using the “MASS” package [78]. Before the proportional odds regression was performed, the muscle and fat scores were pooled into one of three levels “0”, “1” and “>1”, so as to distinguish between birds with minimal, low and higher reserves. Pooling together birds with fat score >1 increased the sample size for this category, allowing more powerful contrasts to be made. The ordinal logistic regression analysis generated an odds ratio for each species in a particular habitat. The odds ratio represents the odds of a particular species having a muscle or fat score one unit higher than a reference species, in a particular habitat.

## Results

### Invertebrate prey resource differences between and within habitats

**Figure 1:**
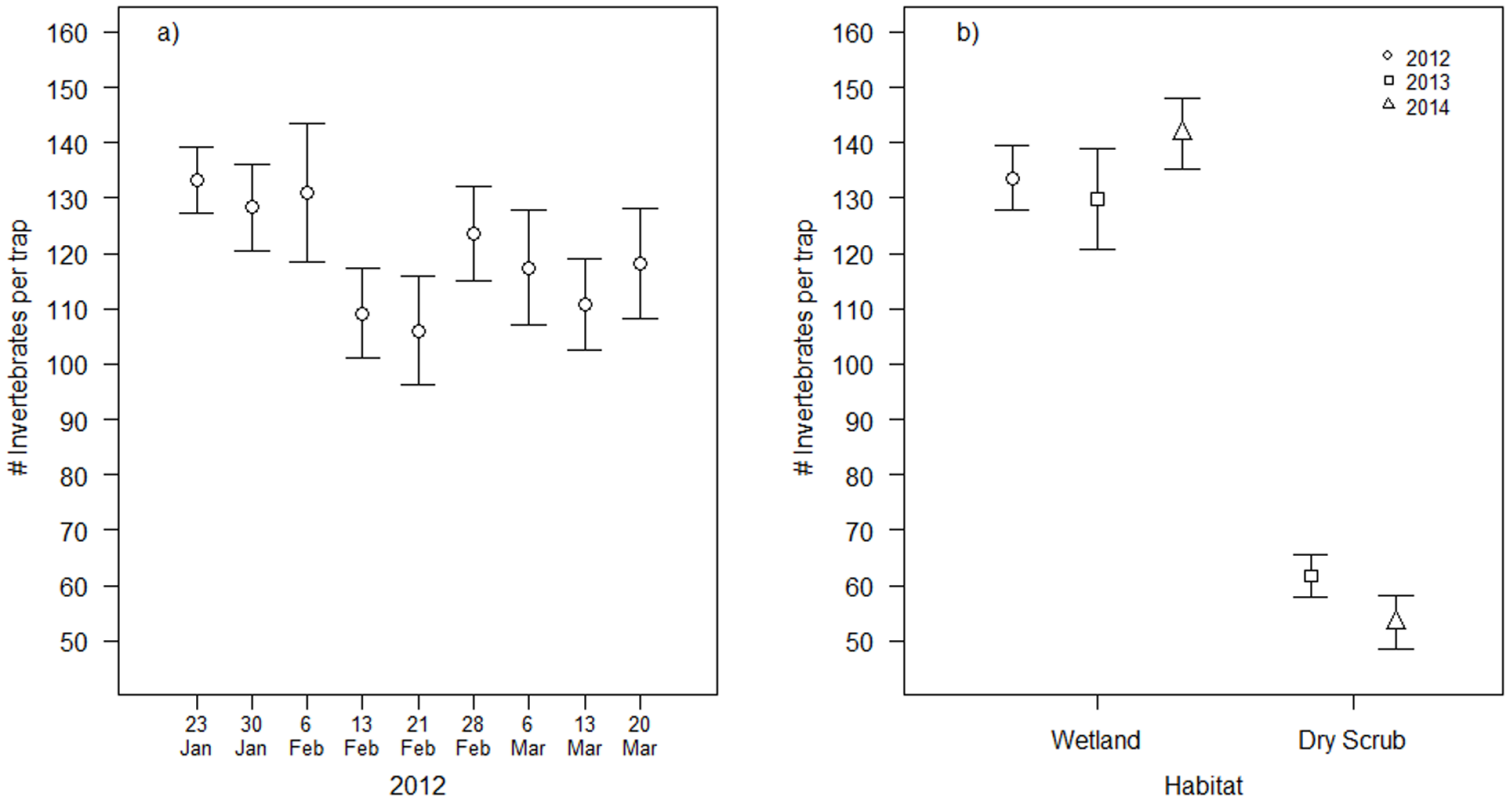
a) Mean numbers of invertebrates per trap (±SE) within wet habitat between 23 January-19 March 2012; and 1b) Wet habitats and dry habitats between 23^rd^–29^th^ January 2012, 22^nd^–30^th^ January 2013, and 21^st^–27^th^ January 2014.

The longer monitoring period in 2012 (three months) revealed a decline of 10.6% in invertebrate abundance over the winter in wetland habitats (GLMM; slope estimate * = −0.017±0.002, Z = −7.36, P<0.0001*; Figure 1a). In both 2013 and 2014, wetland and dry scrub habitats supported a similar invertebrate community at the Order level (Table 2), but mean abundance of invertebrates per sample in the wetlands (*135.2±4.15*) was more than double that of the dry scrub habitat (*57.52±3.07*; GLM: *F_141_,_140_, = 182.141, P<0.0001*; Figure 1b). Variances of invertebrate samples in wetlands were significantly higher than dry scrub in 2013 (*F_27,27_ = 5.891, P<0.0001*) but not in 2014 (*F_29,27_ = 1.88, P = 0.103*). There was significant variation in the occurrence of large invertebrates between wetland and dry scrub habitats (GLM; *F_1,140_ = 37.369, P<0.0001*). Large invertebrates comprised 2.3% of all individuals trapped in wetlands in 2013 and 2014, consisting of Arachnidae, Diptera, Hymenoptera, Lepidoptera (and unidentifiable specimens), compared with 1.1% in dry scrub habitats consisting of Arachnidae, Diptera and Hymenoptera (Table 2).

**Table 2:** Abundances of small and large invertebrates by taxonomic group trapped in wetland and dry scrub sites in Parc National des Oiseaux du Djoudj during January 2013 and 2014 combined.

	Wetlands			Dry Scrub		
	Small	Large		Small	Large	
	(≤5 mm)	(>5 mm)	*n*	(≤5 mm)	(>5 mm)	*n*
Arachnidae	1	25	26	0	10	10
Diptera	6970	41	7011	2124	20	2144
Hymenoptera	3794	19	3813	496	6	502
Hemiptera	307	0	307	54	0	54
Lepidoptera	0	2	2	0	0	0
Coleoptera	34	0	34	144	0	144
Unknown	431	3	434	367	0	367
Total	11364	263	11627	3185	36	3221

### Testing for species differences in habitat preferences of warblers

Over the course of the study, 116 Eurasian reed warblers, 89 subalpine warblers and 194 chiffchaffs were caught across wetland and dry scrub habitats (Table S1). A total of 151 sedge warblers and 67 African reed warblers were caught in wetlands, and 25 olivaceous warblers were caught in dry scrub habitats. There was no significant variation in habitat preference between generalist species (GLM; *F_5, 381_ = 0.177, P = 0.971*) with birds caught in the wetlands (in 2013 and 2014) representing 46.4% of all captures of Eurasian reed warblers, 43.3% of chiffchaffs and 17.9% of subalpine warblers.

### Warbler Body Mass

**Figure 2:**
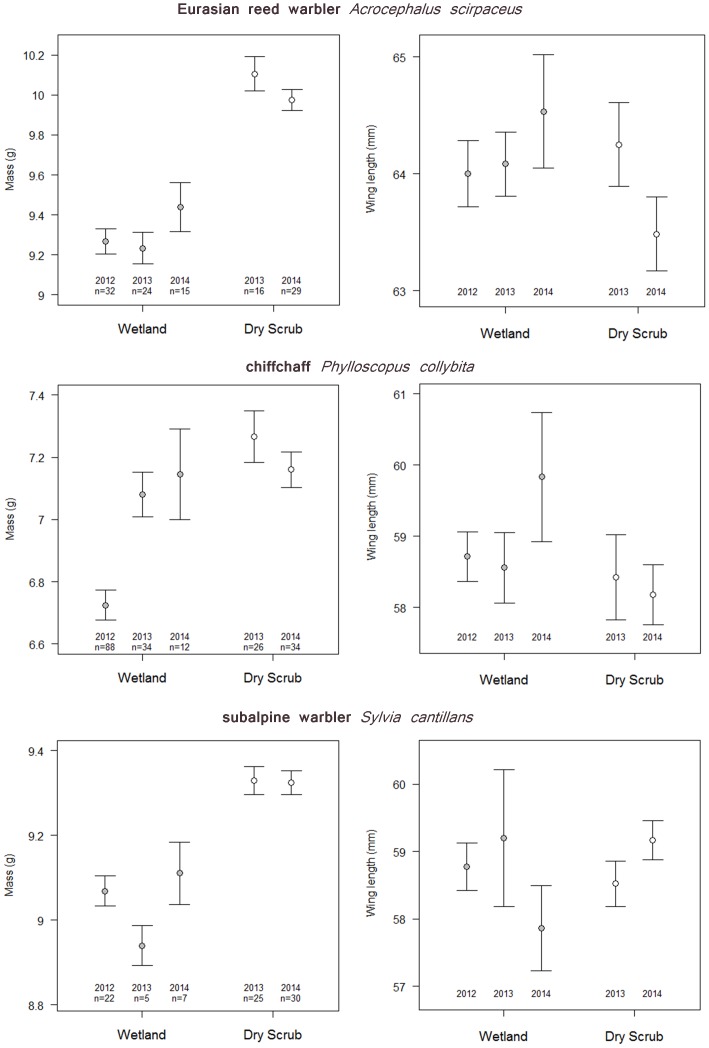
Body mass (± SE, corrected for structural body size and time of day) and wing length (± SE) of wintering populations of generalist warbler species occupying both wetland and dry scrub habitats.

**Figure 3:**
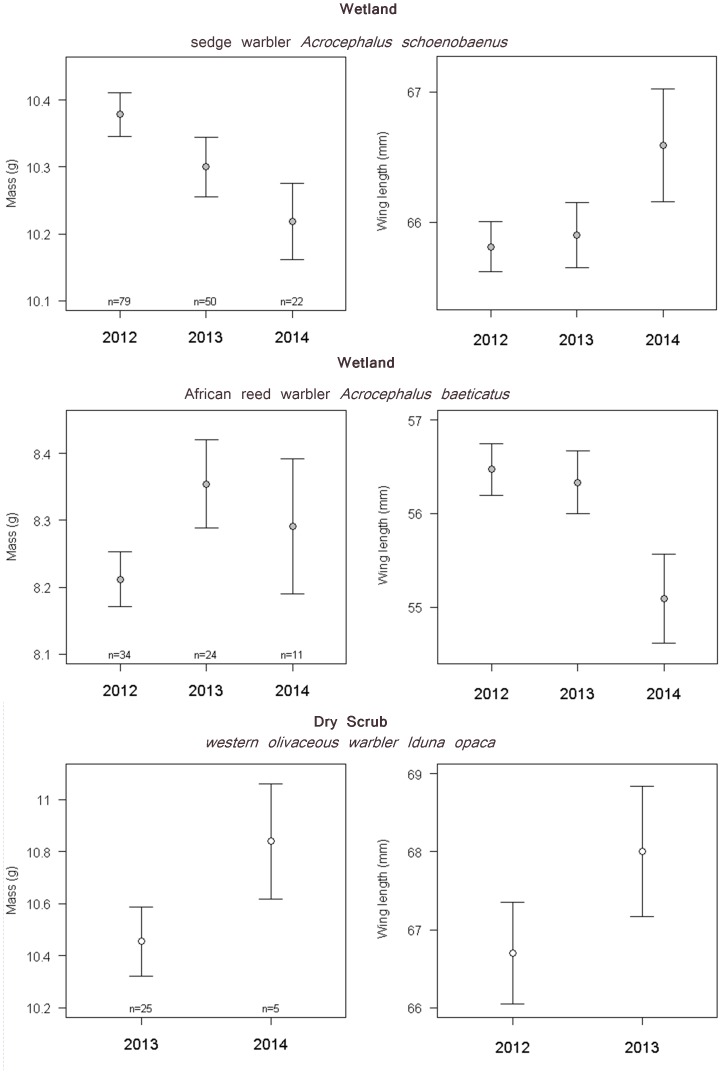
Body mass (± SE, corrected for structural body size and time of day) and wing length (± SE) of wintering populations of habitat specialist warblers occupying either wetland or dry scrub habitats.

Time of day was an important determinant of body mass for all species, with a significant increase in mass from the early morning to reach a peak in the late afternoon (*F*_7.874,8.667_ = 4.872, *P*<0.0001). All else being equal, the estimated mean difference between birds caught at the beginning of the day and those caught in the late afternoon was greatest for Eurasian reed warblers (1.58 g, representing 15.6% of mean winter body mass) and least for chiffchaff (0.49 g, representing 7.0% of mean winter body mass). After controlling statistically for time of day, year and species effects, mass was positively associated with wing length (GAM; 0.089±0.009 g/mm, *t* = 10.495, *P*<0.0001), muscle score (GAM; 0.146±0.049 g/integer, *t* = 2.971, *P* = 0.0031) and fat score (GAM; 0.355±0.021 g/integer, *t* = 16.582, *P*<0.0001). The effect of habitat type differed between species (Table 3). Eurasian reed warblers and subalpine warblers were significantly heavier in dry scrub than wetlands (by 0.71 g (7.0% of mean winter body mass) ±0.14 g and 0.34 g (4.0% of lean mass) ±0.15 g, respectively). Chiffchaffs also weighed more in scrub on average (by 0.17 g (2.4% of lean mass) ±0.11 g) but the difference was not significant. There was no significant inter-annual variation in body mass for Eurasian reed warbler and subalpine warbler but significant variation between years in chiffchaff with 2014 and 2013 masses significantly greater than 2012 (Table 3; Figure 2). Controlling statistically for time of day, body size and the extent of fat and muscle, chiffchaff mass was strongly positively associated with the Sahel precipitation index for the preceding wet season (GAM: +0.20 g ± 0.04 g, *t* = 5.764, *P*<0.0001), indicating greater mass following higher rainfall. Of the habitat specialist species, only African reed warbler showed significant variation in mass, exhibiting an increase in mass between 2012 and 2014 (GAM; +0.399 g±0.177 g, *t* = 2.261, *P* = 0.0274; Figure 3).

**Table 3:** Generalised Additive Models of body mass (dependent variable) for each generalist species. Parameter estimates for levels of the factors “Habitat” and “Year”, are relative to the reference levels of “Wetland” and “2012” respectively.

Species	Parameter	Estimate	SE	z	*d.f.*	*P*
*Eurasian reed warbler*	**Time(s)**	**-**	**-**	***F = 4.658***	**3.59, 3.91**	**0.002**
	**Habitat Dry**	**0.708**	**0.137**	***t = 5.157***		**<0.001**
	**Wing**	**0.131**	**0.037**	***t = 3.575***		**<0.001**
	Total head length	0.131	0.090	*t = 1.257*		0.211
	Tarsus	0.132	0.079	*t = 1.671*		0.098
	Year 2013	−0.193	0.016	*t = −1.207*		0.230
	Year 2014	−0.106	0.180	*t = −0.591*		0.556
*chiffchaff*	**Time(s)**	**-**	**-**	***F = 3.665***	**1.85, 2.18**	**0.024**
	Habitat Dry	0.166	0.113	*t = 1.470*		0.143
	**Wing**	**0.123**	**0.016**	***t = 7.517***		**<0.001**
	Total head length	−0.081	0.065	*t = −1.238*		0.217
	Tarsus	0.090	0.055	*t = 1.633*		0.104
	**Year 2013**	**0.471**	**0.118**	***t = 3.995***		**<0.001**
	**Year 2014**	**0.387**	**0.144**	***t = 2.684***		**0.008**
*subalpine warbler*	**Time(s)**	**-**	**-**	***F = 4.601***	**1.57, 1.89**	**0.012**
	**Habitat Dry**	**0.346**	**0.153**	***t = 2.258***		**0.027**
	Wing	0.028	0.038	*t = 0.742*		0.460
	Total head length	−0.004	0.066	*t = −0.055*		0.956
	Tarsus	−0.001	0.089	*t = −0.012*		0.991
	Year 2013	−0.159	0.193	*t = −0.825*		0.412
	Year 2014	−0.169	0.186	*t = −0.909*		0.366

Effects shown in bold are significant (*P<0.05).*

### Warbler Structural Body Size

There were no significant differences in structural body size between wetland and dry scrub samples of any of the three generalist species, nor were there any significant differences in size between years (Table 4, Figure 3).

**Table 4:** General Linear Model of structural body size (dependant variable) for each generalist species.

Species	Parameter	Estimate	SE	*t*	d.f.	*P*
*Eurasian reed warbler*	Habitat Dry	−0.451	0.3652	−1.235	112	0.219
	Year 2013	0.330	0.409	0.807		0.421
	Year 2014	0.138	0.445	0.310		0.757
*chiffchaff*	Habitat Dry	−0.707	0.629	−1.125	190	0.262
	Year 2013	0.091	0.580	0.156		0.876
	Year 2014	0.416	0.725	0.573		0.567
*subalpine warbler*	Habitat Dry	0.467	0.544	0.859	85	0.393
	Year 2013	−0.529	0.659	−0.802		0.425
	Year 2014	−0.233	0.637	−0.365		0.716

Parameter estimates for levels of the factors “Habitat” and “Year”, are relative to the reference levels of “Wetland” and “2012” respectively.

An equivalent model using 2013 and 2014 data only (excluding 2012 on the grounds that there was no habitat contrast available) showed there was no significant year x habitat interaction influencing body size in Eurasian reed warblers (*t_83_* = −1.683, *P* = 0.0963), chiffchaff (*t_105_* = −1.264, *P* = 0.209) or subalpine warbler (*t_66_* = 1.824, *P* = 0.0729), despite some apparent size differences between 2013 and 2014 in Figure 2. Of the habitat specialist species, only African reed warbler showed significant differences in the population wing length, with birds in 2014 being 1.38±0.561 mm smaller than birds in 2012 (GLM; *F*_68,66_ = −3.142, *P* = 0.0497; Figure 3).

### Body reserves

**Figure 4:**
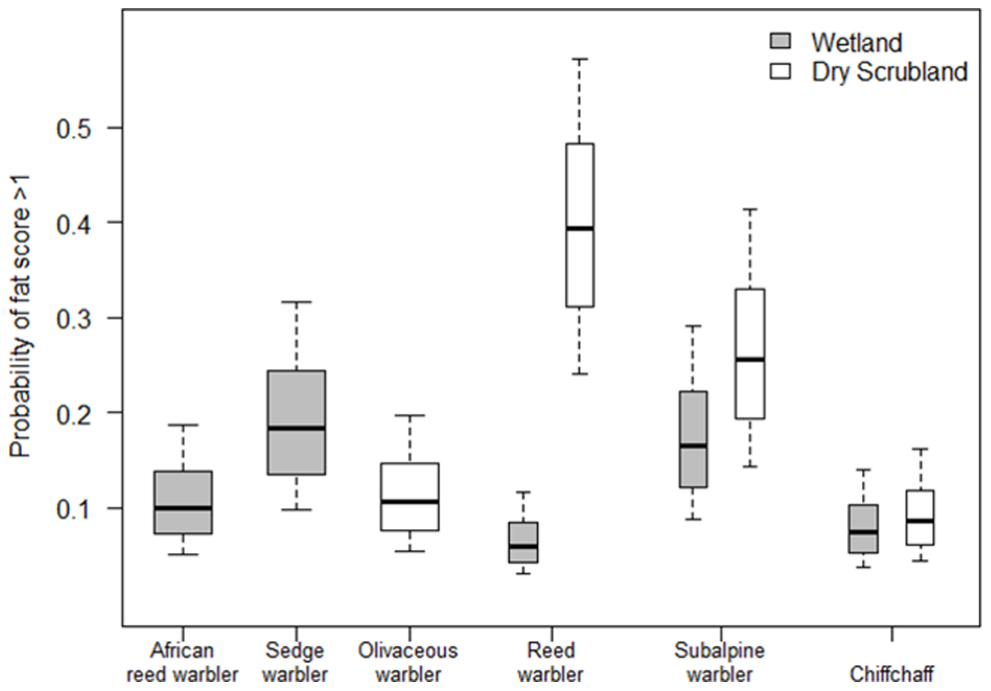
Model predictions for the probability of observing birds in wetlands or dry scrub habitat with a fat score of greater than 1.

The proportional odds regression revealed that muscle score was not significantly affected by habitat type (*t* = 0.492, *P* = 0.6224) or time of day (*t* = 1.432, *P* = 0.1521). The ordinal logistic regression analysis generates an odds ratio for each species in a particular habitat, which represent the odds of having a muscle score one unit higher than a reference species, in a particular habitat. The only interspecific difference was the wetland specialist species African reed warbler having 1.50 times greater odds (*P* = 0.035) of having a muscle score one unit higher than the dry- scrub specialist western olivaceous warbler (Table 5). Fat score was significantly affected by habitat type (*t* = −3.944, *P*<0.0001; Table 5, Figure 4) and time of day (*t* = 3.874, *P* = 0.0001). Eurasian reed warblers had 2.32 times greater odds of having a fat score one unit higher in the dry-scrub than in the wetlands (Table 6; Figure 4). The wetland specialist species sedge warbler had 1.26 times greater odds of having a fat score one unit higher relative to Eurasian reed warbler occupying the wetland habitat. Conversely, in the dry habitats, Eurasian reed warbler had 1.70 times greater odds of having a fat score one unit higher relative to the dry habitat specialist olivaceous warbler.

**Table 5:** Pairwise comparison of proportional odds of species in a particular habitat having a muscle score of 1 unit higher than another species in a particular habitat, or the same species in a different habitat.

		Wetland Habitat				Dry Scrub Habitat			
		Subalpine Warbler	Chiffchaff	Sedge Warbler	A. Reed Warbler	E. Reed Warbler	Subalpine Warbler	Chiffchaff	Olivaceous Warbler
Wetland Habitat	E. Reed Warbler	−0.82	0.06	−0.88*	−0.74	−0.46	−0.32	0.48	0.29
	Subalpine Warbler		0.88	−0.06	−0.38	0.36	0.49	1.30*	1.12
	Chiffchaff			−0.94**	−1.26	−0.52	−0.39	0.42	0.24
	Sedge Warbler				−0.32	0.42	0.55	1.36**	1.17
	A. Reed Warbler					0.74	0.87	1.68**	1.50*
Dry Scrub Habitat	E. Reed Warbler						0.13	0.94	0.75
	Subalpine Warbler							0.81	0.62
	Chiffchaff								−0.19

Displayed odds values relate to species in the vertical column in reference to those in the horizontal row above the matrix. Positive odds values indicate a higher likelihood, and negative odds indicate a lower likelihood. The sign would be reversed to obtain the odds value for the species groups in the horizontal row in reference to those in the vertical column. * = P<0.05, **  = P<0.005, *** = P<0.0001.

**Table 6:** Pairwise comparison of proportional odds of species in a particular habitat having a fat score of 1 unit higher than another species in a particular habitat, or the same species in a different habitat.

		Wetland Habitat				Dry Scrub Habitat			
		Subalpine Warbler	Chiffchaff	Sedge Warbler	A. Reed Warbler	E. Reed Warbler	Subalpine Warbler	Chiffchaff	Olivaceous Warbler
Wetland Habitat	E. Reed Warbler	−1.14*	−0.22	−1.26**	−0.56	−2.32***	−1.68**	−0.39	−0.63
	Subalpine Warbler		0.91	−0.12	0.58	−1.18*	−0.55	0.75	0.51
	Chiffchaff			−1.04**	−0.34	−2.10***	−1.46***	−0.17	−0.40
	Sedge Warbler				0.7	−1.06**	−0.42	0.87*	0.63
	A. Reed Warbler					−1.76***	−1.12*	0.17	−0.07
Dry Scrub Habitat	E. Reed Warbler						0.64	1.93***	1.70*
	Subalpine Warbler						1.29*	1.06	1.29*
	Chiffchaff								−0.23

Displayed odds values relate to species in the vertical column in reference to those in the horizontal row above the matrix. Positive odds values indicate a higher likelihood, and negative odds indicate a lower likelihood. The sign would be reversed to obtain the odds value for the species groups in the horizontal row in reference to those in the vertical column. * = P<0.05, **  = P<0.005, *** = P<0.0001.

## Discussion

As expected, wetland habitats supported consistently greater invertebrate resources than dry scrub habitats [79]–[82]. On the basis of food availability, wintering generalist warblers should therefore favour wetlands over the dry scrub habitats. Eurasian reed warblers occupied both habitats and carried substantially larger body reserves in the dry scrub habitat compared to those occupying the wetland habitat, consistent with greater strategic buffering of energy reserves against starvation in the dry scrub habitat where starvation risk is greatest.

Starvation risk is a function of both the overall (mean) level of food availability, and its variance. Eurasian reed warblers carried higher body reserves in dry scrub than wetland in both years when this comparison was made, including 2013 when variance in prey availability was higher in wetland habitats than dry scrub but the mean abundance remained approximately twice as high in the wetland. This indicates that even the lower end of the range of prey availability in wetlands in 2013 was sufficient to allow birds to maintain low fat reserves without an excessive risk of starvation.

Although muscle was a significant predictor of body mass in this study, the effect-size was low muscle score differed less than fat between habitats. This pattern supports the concept that adaptive changes to mass are likely to involve dynamic fat reserves while loss of functional muscle is more likely to be a consequence of physiological stress [83]. It is also possible that the method of muscle measurement (a three-point scale) provides insufficient resolution to detect a difference between habitats in muscle score. Similarly, the extent to which visible fat scores reflect the relative body-lipid reserves may limit conclusions about adaptive fat deposition [76]. There were no significant differences in structural body size of reed warblers between habitats or years and, since we were unable consistently to determine the age and sex of the warblers in our study, we were not able to test for intra-specific differences (e.g. age and sex variation) in habitat effects as found in several other studies of migrants in their wintering habitats [4], [31], [84], [85]. It is thus possible that the distribution of individuals is a result of age or sex-biased habitat segregation, despite the absence of any significant differences in wing length between habitats in our dataset.

These results suggest that mass is regulated strategically among warblers overwintering in the Sahel, rather than being limited directly by food availability. Experimental manipulations of food availability and predictability have shown that birds can strategically increase mass through fat accumulation to buffer against energetic shortfalls [35], [38], [40], [42]. A similar effect is evident in the context of migratory fuelling, where larger energetic reserves are accumulated in anticipation of low food availability ahead of large ecological barriers [86].

Although higher energy reserves are better for avoiding starvation and for fuelling migration, the extent of muscle and fat accumulation must be balanced against the costs of maintaining higher body mass, such as increased wing-loading costs on flight performance and longer foraging periods, both of which increase susceptibility to predation [87]. The differences in predator density across wetland and dry scrub habitats in this study are unknown but it is likely that birds inhabiting the dry scrub habitats would have higher exposure to predators as they must more frequently cross open spaces between habitat patches than in the wetlands. Subalpine warblers and chiffchaffs exhibited similar but less pronounced habitat differences in body reserves than observed in the Eurasian reed warbler (though this habitat difference was non-significant in chiffchaffs). Although subalpine warblers and chiffchaffs were regularly captured in wetlands, it is notable that these captures occurred mainly in the scrub-*Phragmites* interface, rather than in the pure stands of *Phragmites* where Eurasian and African reed warblers and sedge warblers were all regularly captured. This is consistent with the less pronounced habitat difference in body reserves in subalpine warblers and chiffchaffs being due to their more limited exploitation of the invertebrate-rich *Phragmites* stands compared to Eurasian reed warblers and the two wetland-specialist species.

The significantly lower mass for chiffchaffs in 2012 compared with 2013 and 2014 may be due to direct food limitation following the low rainfall (as measured by the Sahel precipitation index) recorded in the preceding wet season, of 2011. This response is contrary to the strategic buffering strategy expected for birds in food-reduced habitats, suggesting direct food limitation in drought conditions. This association with Sahel precipitation index was not observed in the other species in this study, although studies of survival of trans-Saharan migrants indicate that drought can directly limit survival. For example, Sahel rainfall has been found to be significantly associated with survival in the sedge warbler [16], [88], reed warbler [89], sand martin [90], [17] and common nightingale *Luscinia megarhynchos* [91], indicating that drought can impair survival, presumably via the mechanism of food limitation.

We found that habitat specialist species maintained lower energetic reserves than generalist species in their preferred habitats. This was the case for African reed warbler and western olivaceous warbler, but not for sedge warbler. The analysis suggested that sedge warblers were more likely to carry higher fat levels than any other species in the wetland habitat. A possible testable explanation for this is that sedge warblers rely on a narrower range of prey than Eurasian and African reed warblers, with a foraging strategy adapted to exploiting slow moving or sedentary prey [92]. Although such adaptation is beneficial during seasonal super-abundances of such prey in the pre-migratory grounds [93]–[95], it may restrict their wintering habitat to wetlands and limit their foraging time to the cooler parts of the day when active aerial invertebrates are easier to catch. Such limitations may require the accumulation of fat reserves despite the occupation of invertebrate-rich wetland habitat. Another important consideration in the interpretation of these results is that the data were collected during the middle of the dry season, when wetlands have undergone contraction since the time of migrant arrival at the end of the wet season [5], [96], [97]. It is therefore possible that the availability of prey suitable for sedge warblers is particularly low at this time of year. Scarcity of certain prey taxa may explain why sedge warbler populations are particularly vulnerable in the winters following low wet-season rainfall [16], [88]. In contrast to sedge warblers, Eurasian reed warblers do not suffer such dramatic population crashes in response to Sahelian drought events [49], [98], [99]. Our results suggest that this may be due to their ability to exploit invertebrate resources in dry habitats in addition to the high quality but intensely competitive and drought-prone wetland habitats.

Although our results suggest an explanation of the presence of different species in a particular habitat or multiple habitats by their ability to exploit resources and survive in these habitats, we are not yet able to explain the process by which community assembly occurs. It is conceivable that habitat choice may be determined by order of arrival, with earliest arriving migrants (e.g. sedge warblers) occupying the highest quality habitats, while later arriving migrants (e.g. Eurasian reed warbler) must select the secondary habitats. However, this may be unlikely considering the findings of studies of interspecific territorialism and competition for space between reed and sedge warblers on the breeding grounds [100], [101] in which early arriving sedge warblers are often displaced by the later arriving reed warblers.

In conclusion, the observed patterns of habitat occupation and body condition described in our study are consistent with the hypothesis that when individuals are in food-poor habitats, they utilise strategic buffering to avoid starvation. This mechanism may only be effective above a critical food availability threshold. Below this threshold, bird mass is likely to be directly limited by food availability [30], [84], [85]. Although there was little evidence for such direct limitation of body reserves during our study period (other than among chiffchaffs), such patterns of nutritional stress are observed in wintering sand martins *Riparia riparia* [17] and barn swallows *Hirundo rustica* [102] in the Sahel during drought years and are implicated as a major cause of population-level declines through increased mortality and carry over effects on productivity [103]–[105]. Similarly, impacts of physiological stress have been shown in other contexts, such as in American redstarts *Setophaga ruticilla* [31], [85] northern waterthrush *Seiurus noveboracensus* [60] and ovenbirds *S. aurocapillus* [30], [106] occupying low quality non-breeding habitats in the Neotropics. Such studies suggest that if foraging conditions in the Sahel become worse as a result of climate-driven and anthropogenic habitat degradation, migrant warblers may become increasingly constrained in their ability to use strategic buffering to reduce the threat of starvation and survive the winter.

## Supporting Information

Table S1 **Number of birds captured in each habitat in January 2012 (wet only), 2013 and 2014.**
(DOCX)
