# Supplementary material for: Habitat Use and Body Mass Regulation among Warblers in the Sahel Region during the Non-Breeding Season
Source: PLoS One. 2014 Nov 26;9(11):e113665. doi: 10.1371/journal.pone.0113665 (PMC4245214; doi:10.1371/journal.pone.0113665)
Supplement: Table S1 — Number of birds captured in each habitat in January 2012 (wet only), 2013 and 2014. (DOCX) [file pone.0113665.s001.docx]

| **Species** | **Year** | **Wet** | **Dry** | **% wet (2013-14)** |
| --- | --- | --- | --- | --- |
| *Eurasian reed warbler* | 2012 | 32 | - |  |
|  | 2013 | 24 | 16 | 46.4 |
|  | 2014 | 15 | 29 |  |
| *common chiffchaff* | 2012 | 88 | - |  |
|  | 2013 | 34 | 26 | 43.3 |
|  | 2014 | 12 | 34 |  |
| *subalpine warbler* | 2012 | 22 | - |  |
|  | 2013 | 5 | 25 | 17.9 |
|  | 2014 | 7 | 30 |  |
| *sedge warbler* | 2012 | 79 | - |  |
|  | 2013 | 50 | 0 | 98.6 |
|  | 2014 | 22 | 1 |  |
| *African reed warbler* | 2012 | 34 | - |  |
|  | 2013 | 24 | 0 | 91 |
|  | 2014 | 11 | 3 |  |
| *western olivaceous warbler* | 2012 | 0 | - |  |
|  | 2013 | 0 | 20 | 0 |
|  | 2014 | 0 | 5 | 0 |
